# Supplementary material for: WordCommentsAnalyzer: A windows software tool for qualitative research
Source: F1000Res. 2018 Sep 4;7:536. Originally published 2018 May 3. [Version 2] doi: 10.12688/f1000research.14819.2 (PMC6137411; doi:10.12688/f1000research.14819.2)
Supplement: Supplementary file 3 [file f1000research-7-17614-s0002.tgz › b2a79496-4ada-47c4-9682-a99ce867836d.zip!/Eight Iranian Health Journals/Journal of Research in Rehabilitation Sciences _39.docx]

© Journal of Research in Rehabilitation Sciences . This work is licensed under CC BY-NC 4.0 (https://creativecommons.org/licenses/by-nc/4.0/).

Volume & Issue: دوره 8، شماره 2: 1391

Title: تعیین پایایی بازآزمایی و پایایی بین ارزیابان نسخه فارسی سیستم طبقه‌بندی توانایی دستی (Manual ability classiﬁcation system) کودکان فلج مغزی

http://jrrs.mui.ac.ir/index.php/jrrs/article/view/422

مقدمه: تعيين ميزان توانايي كودكان فلج مغزي در برنامه‌ريزي درمان و ارايه خدمات حمايتي توسط سازمان‌هايي همچون بهزيستي نقش اساسي و مهمي را بازي مي‌كند. سیستم طبقه‌بندی توانایی دستی (Manual ability classiﬁcation system یا MACS) قسمتي از اين مهم را با طبقه‌بندي توانایي دستي كودكان به انجام مي‌رساند. اين سيستم طبقه‌بندي در ايران به فارسي برگردانيده شده است، ولي نياز به مطالعه بيشتر دارد. بنابراین هدف از این پژوهش تعیین پایایی تکرار آزمون و پایایی بین ارزیابان نسخه فارسی سیستم MACS از عملکرد دست کودکان فلج مغزی (Cerebral palsy یا CP) بود. مواد و روش‌ها: جهت تعیین پایایی نسخه فارسی MACS، پایایی بازآزمایی و پایایی بین ارزیابان، ابتدا 39 کودک 18-4 سال با میانگین سنی 93/12 بر حسب ماه (16 دختر و 23 پسر با انواع مختلف فلج مغزی) در پنج سطح MACS انتخاب شدند. سپس از هر کودک در حین اجرای 8 فعالیت روزمره مثل غذا خوردن، فیلم تهیه گردید. در نهایت 6 کاردرمانگر با مشاهده این فیلم‌ها سطح MACS را برای هر کودک مشخص نمودند. این امر در دو نوبت به فاصله زمانی چهار هفته تکرار گردید. جهت تجزیه و تحلیل آماری از ضریب همبستگی درون خوشه‌ای (ICC  یا Interclass correlation coefficient) و ضریب کاپای وزنی در نرم‌افزار 16SPSS استفاده گردید. همچنين سطح معنی‌داري معادل 0/05 ≤ P در نظر گرفته شد. یافته‌ها: پايايي بين ارزيابان بسيار بالا بود؛ به گونه‌اي كه ضریب همبستگی درون خوشه‌ای (ICC) بین 6 کاردرمانگر در نوبت اول (0/96) و در نوبت دوم (0/95) بالای 0/9 به دست آمد و ضریب کاپای وزنی نیز بالای 0/75 بود که هر دو نشان دهنده میزان توافق عالی است. برای پایایی بازآزمایی، ICC در مورد درمانگران 0/98 به دست آمد و همچنین ضریب کاپای وزنی نیز بالای 0/75 بود که هر دو بیانگر میزان همبستگی بالا بوده‌اند. نتیجه گیری: نسخه فارسی مقیاس MACS از پایایی تکرار آزمون و پایایی بین ارزیابان کافی برخوردار است و می‌توان از آن به عنوان ابزاری مفید و کاربردی در ارزیابی میزان توانایی دستی کودکان فلج مغزی استفاده نمود.

Title: انتخاب فهرست برتر در آزمون بازشناسی لغات در افراد با شنوایی طبیعی

http://jrrs.mui.ac.ir/index.php/jrrs/article/view/343

مقدمه: ادیومتری گفتاری یکی از ضروری‌ترین اجزای مجموعه آزمون‌های شنوایی است که اطلاعاتی را در ارتباط با حساسیت شنوایی فرد و فهم گفتار در شدت‌های فوق آستانه فراهم می‌کند. با توجه به اهمیت آزمون امتیاز بازشناسی لغات و عدم داشتن فهرست یکسان جهت استفاده بالینی، در اين تحقيق سعی بر آن شد كه 4 فهرست پرکاربرد در بزرگسالان با شنوايي طبيعي مورد بررسی قرار گیرد تا در صورت احراز نتایج مورد نظر، فهرستی به عنوان فهرست برتر انتخاب شود. مواد و روش‌ها: این مطالعه به روش توصیفی- تحلیلی بود. جمعیت مورد مطالعه افراد 40-18 سال، شامل 30 فرد با شنوایی طبیعی بود. آزمون با استفاده از چهار فهرست انتخابی (2، 4، 5 و 9) انجام پذیرفت. یافته‌ها: در افراد با شنوایی طبیعی امتیازهای هر چهار فهرست در محدوده 100-92 درصد به دست آمد. در افراد با شنوایی طبیعی میانگین امتیاز در فهرست 2، 99/65 درصد؛ در فهرست 4، 94/13 درصد؛ در فهرست 5، 98 درصد و در فهرست 9، 98/53 درصد بود. بر اساس امتیازدهی لغتی در گروه هنجار فهرست 9 بیش‌ترین پایایی را دارا بود. نتیجه گیری: با توجه به این که همه فهرست‌ها از پایایی بالایی برخوردار هستند، اما با در نظر گرفتن تفاوت‌های جزیی در پایایی لغتی و واجی و همچنین هم ارزی فهرست‌ها، فهرست 9 به عنوان فهرست برتر معرفی گردید.

Title: تأثیر تمرینات ورزشی در حين دياليز بر خستگی و كيفيت زندگي بيماران همودياليزي

http://jrrs.mui.ac.ir/index.php/jrrs/article/view/445

مقدمه: درمان همودیالیز در دراز مدت زندگی بیمار را تحت تأثیر قرار می‌دهد. بیشتر بیماران همودیالیزی سبک زندگی غیر فعال دارند که عوارضی را برای آن‌ها به دنبال دارد. از این ‌رو هدف از مطالعه حاضر، تعیین تأثیر تمرينات ورزشي در بهبود كيفيت زندگي و قدرت عضلانی این گروه بیماران بود. مواد و روش‌ها: این مطالعه به صورت تجربی به روش پیش آزمون- پس آزمون با گروه تجربی و شاهد انجام شد. بدین منظور، 30 بیمار تحت درمان همودیالیز با میانگین سنی 45 سال و سه بار دیالیز در هفته در مرکز دیالیز شریعتی اصفهان به صورت داوطلب انتخاب شدند و به صورت تصادفی در دو گروه تجربی و شاهد قرار گرفتند. داده‌ها به وسیله پرسش‌نامه کیفیت زندگی (Kidney disease quality of life-short form یا KDQOL-SFtm) و آزمون نشستن و برخاستن از روی صندلی جمع‌آوری گردید. تمرینات ورزشی با استفاده از دوچرخه در حین دیالیز به مدت 5 ماه، 3 جلسه در هفته، هر جلسه حدود 60 دقیقه برای گروه تجربی طراحی شد. در طی این مدت، گروه شاهد در هیچ گونه فعالیت ورزشی شرکت نکردند. در این مطالعه از روش‌های آماری توصیفی و استنباطی ANCOVA جهت تجزیه و تحلیل اطلاعات در بسته نرم‌افزاری 18SPSS استفاده شده است. یافته‌ها: کیفیت زندگی گروه تجربی پس از 5 ماه تمرینات ورزشی بهبود معنی‌داری داشته است (0/05 < P). در صورتی که بهبودی در گروه شاهد معنی‌دار نبود (0/05 > P). همچنین قدرت عضلانی در گروه تجربی (0/05 < P) نسبت به گروه شاهد (0/05 < P) افزایش معنی‌داری را نشان داد (0/05 > P). نتیجه گیری: نتایج این مطالعه نشان داد که تمرینات ورزشی بر کیفیت زندگی و رفع خستگی و بهبود قدرت عضلات بیماران همودیالیزی تأثیر داشته است. با توجه به نتایج، اهمیت تمرینات ورزشی در حین دیالیز برای ارتقای بعد سلامت جسمی و روحی در مراکز همودیالیز روشن می‌گردد.

Title: مقایسه تأثير تمرين ذهني و تمرين بدني بر يادگيري برنامه حرکتي تعميم يافته و پارامتر مهارت پيگردي چرخان

http://jrrs.mui.ac.ir/index.php/jrrs/article/view/440

مقدمه: هدف تحقيق حاضر بررسي و مقايسه تأثير تمرين ذهني و تمرين بدني بر يادگيري برنامه حرکتي تعميم يافته و پارامتر مهارت حرکتي تعقيبي بود. مواد و روش‌ها: در این مطالعه نیمه تجربی، آزمودني‌ها که شامل 20 نفر از دانشجويان کارشناسي دانشگاه شهيد بهشتي بودند به طور داوطلبانه در تحقيق شرکت کردند. بعد از پيش‌ آزمون، آزمودني‌ها مهارت پيگردي چرخان را در يک دوره سه جلسه‌اي به صورت یک روز در ميان تمرين کردند و بعد از 48 ساعت استراحت براي آزمون يادداري فرا خوانده شدند. براي تحليل داده‌ها از آزمون‌هاي Dependent t و Independent t توسط نرم‌افزار 16SPSS در سطح معني‌داري 0/05 استفاده شد. یافته‌ها: استفاده از هر دو روش تمرين ذهني و تمرين بدني يادگيري هر دو جزء برنامه حرکتي و پارامتر مهارت پيگردي را به صورت معني‌داري افزايش داد (0/001 = P). همچنين يافته‌هاي تحقيق بين تأثير تمرين ذهني و تمرين بدني به لحاظ اثرگذاري بر يادگيري برنامه حرکتي تعمیم یافته و پارامتر مهارت پيگردي تفاوت معني‌داري نشان نداد (0/05 ≤ P). نتیجه گیری: تمرين ذهني مي‌تواند جايگزين مناسبي براي تمرين بدني به هنگام بازتواني‌ و يادگيري دو جزء برنامه حرکتي تعميم يافته و پارامتر يک مهارت حرکتي پيگردي باشد، به ويژه در شرايطي که فرد نمي‌تواند از اندام خود براي اجراي مهارت و فعاليت بدني استفاده کند.

Title: مقایسه سرعت حرکتی و برتری جانبی در کودکان و نوجوانان مبتلا به لکنت رشدی و همتایان سالم

http://jrrs.mui.ac.ir/index.php/jrrs/article/view/318

مقدمه: لكنت اختلال شايعي در ميان كودكان و نوجوانان مي‌باشد. هدف از این مطالعه مقایسه سرعت حرکتی و برتری جانبی در کودکان و نوجوانان مبتلا به لکنت رشدی و همتایان سالم بود. مواد و روش‌ها: این پژوهش از نوع مقطعی- تحلیلی بود. با روش نمونه‌گیری در دسترس و به كمك تشخیص يك گفتار درمانگر، 30 دانش‌آموز مبتلا به لكنت رشدي و 30 دانش‌آموز نيز به عنوان همتايان سالم از مدارس مقاطع ابتدایی و راهنمایی شهریار انتخاب گرديد. هر گروه شامل 26 پسر و 4 دختر بود و میانگین سنی آن‌ها 12 سال به دست آمد. تكليف ضربه زدن با انگشت به عنوان آزمون عصب- روان‌شناختي جهت ارزیابی مورد استفاده قرار گرفت. از آزمون‌های Dependent t و Independent t و نرم‌افزار آماری 18SPSS جهت تجزیه و تحلیل داده‌ها استفاده شد. یافته‌ها: بین دو گروه از نظر سرعت حركتي تفاوت معنی‌داری وجود داشت (0/0001 ≤ P). همچنین برتری جانبی در هر دو گروه الگوی مشابهی داشت (0/35 = P). سرعت حرکتی دست راست (مربوط به نیمکره چپ) برتری معنی‌داری نسبت به سرعت حرکتی دست چپ (مربوط به نیمکره راست) در هر دو گروه داشت. در نتیجه نیمکره چپ در هر دو گروه نیمکره برتر می‌باشد. نتیجه گیری: وقتی تولید زبان آسیب دیده است، مشکلاتی در سرعت حرکتی اندام‌های پیرامونی نیز ظاهر می‌شود. در ارزیابی بالینی و مداخلات مربوط به گروه‌های مبتلا به لكنت باید به همزمانی آسیب‌های زبانی و نقص‌های حرکتی توجه شود. به علاوه، ممکن است برتری جانبی نامعمول پیامد اختلال لکنت و نه بخشی از سبب شناسی آن باشد.

Title: تأثیر یک دوره تمرینات پیلاتس بر دیسمنوره اولیه

http://jrrs.mui.ac.ir/index.php/jrrs/article/view/437

مقدمه: دیسمنوره اولیه یا قاعدگی دردناک بدون حضور عارضه‌ پاتولوژیک لگنی، از شایع‌ترین شکایات در طب زنان می‌باشد. هدف کلی این تحقیق بررسی اثر تمرینات پیلاتس بر دیسمنوره اولیه بود. مواد و روش‌ها: در این مطالعه مداخله‌ای از میان 1500 نفر دانشجوی دختر، 40 دختر 18 تا 25 ساله غیر ورزشکار مبتلا به دیسمنوره اولیه به صورت هدفمند انتخاب و به دو گروه تجربی (20 نفر) و شاهد (20 نفر) تقسیم شدند. جهت جمع‌آوری داده‌ها از پرسش‌نامه سنجش دیسمنوره اولیه استفاده گردید. نمونه‌ها تمرینات پیلاتس را به مدت هشت هفته زیر نظر مربی پیلاتس انجام دادند. براي تجزيه و تحليل داده‌ها از آزمون‌های Shapiro-Wilk، ANOVA با اندازه‌گیری مکرر و t Dependent با تعیین سطح معنی‌داري 0/05 ≤ P استفاده شد. یافته‌ها: بهبود معنی‌داری در شدت درد (0/001 > P) و مدت درد (0/001 > P) در گروه پیلاتس پس از درمان دیده شد. اما از نظر میزان خونریزی (0/18 = P) اختلاف معنی‌داری مشاهده نشد. در گروه شاهد نیز تغییر معنی‌داری در این شاخص‌ها دیده نشد. از طرف دیگر، مقایسه داده‌های دو گروه اختلاف معنی‌داری را در شدت درد (0/001 > P) و مدت درد (0/001 > P) پس از مداخله نشان داد. اما از نظر میزان خونریزی (1/00 = P) بین دو گروه اختلاف معنی‌داری دیده نشد. نتیجه گیری: با توجه به نوع خاص تمرینات پیلاتس، این تمرینات شاید بتواند باعث کاهش شدت و مدت درد دیسمنوره اولیه گردد.

Title: درک تجربه عدم اشتغال در افراد مبتلا به ضایعات نخاعی در روند برگشت به کار

http://jrrs.mui.ac.ir/index.php/jrrs/article/view/378

مقدمه: از دست دادن کار و موانع موجود در راه بازگشت به کار از پیامدهای ابتلا به ضایعات نخاعی است. این مقاله شامل بخشی از نتایج پژوهش کیفی تحت عنوان "درک تجربه زیسته افراد مبتلا به ضایعات نخاعی در روند برگشت به کار در شهر قم" بود که با هدف شناخت عوامل زمینه‌ساز عدم موفقیت در برگشت به کار پس از ابتلا به ضایعه نخاعی تنظیم شد. مواد و روش‌ها: پژوهش فوق به روش کیفی و شیوه پدیده‌ شناسی انجام شد. شرکت کنندگان، مردان مبتلا به ضایعه نخاعی پاراپلژی دارای پرونده در اداره بهزیستی استان قم بودند. نمونه‌گیری به روش هدفمند بود و تا اشباع کامل اطلاعات ادامه یافت. تعداد شرکت کنندگان به 20 نفر رسید که در دو گروه 10 نفری شاغل و غیر شاغل قرار داشتند. جمع‌آوری داده‌ها با استفاده از مصاحبه نیمه ساختار یافته باز عمیق فردی و تفسیر داده‌ها به روش Van Mannen انجام شد. یافته‌ها: در مجموع 6 مضمون محوری در این پژوهش ظهور یافت که بیانگر تجارب شرکت کنندگان از مشکلات و موانع موجود در راه بازگشت به کار بود. نتیجه گیری: بررسی‌ها نشان داد که در برخی زیرمضمون‌ها تفاوت‌هایی در گفته‌های افراد غیر شاغل با شاغلین وجود دارد که بیانگر عوامل تعیین کننده موفقیت یا عدم موفقیت در برگشت به کار بود. در واقع می‌توان گفت درک نادرست افراد از مشکلات جسمی، نوع شخصیت وابسته و غیرفعال، نگرش منفی نسبت به مسایل و فقدان حمایت‌های روحی- روانی خانواده از موانع برگشت به کار اختصاصی افراد غیر شاغل در این مطالعه بود.

Title: اثربخشی آموزش مهارت‌های ارتباط مؤثر بر عزت نفس دختران دچار ناتوانی جسمی- حرکتی

http://jrrs.mui.ac.ir/index.php/jrrs/article/view/475

مقدمه: پژوهش حاضر به منظور بررسی اثربخشی مهارت‌های ارتباط مؤثر بر عزت نفس دختران ناتوان جسمی- حرکتی شهر مشهد انجام شد. مواد و روش‌ها: این پژوهش یک طرح شبه آزمایشی با پیش آزمون- پس آزمون تک گروهی بود. 15 نفر به عنوان آزمودنی شرکت داشتند که با روش نمونه‌گیری در دسترس از مرکز فیاض‌بخش مشهد انتخاب شدند. کلیه شرکت کنندگان که با توجه به مؤلفه سن و جنس و وضعیت تأهل در گروه سنی 15 تا 30 سال، دختر و مجرد بوده‌اند، پرسش‌نامه عزت نفس Cooper-Smith (Cooper-Smith self-esteem inventory یا SEI) را تکمیل نمودند. آزمودنی‌ها به مدت ده جلسه در جلسات آموزش مهارت‌های ارتباط مؤثر گروهی شرکت داشتند و پس از اتمام جلسات آموزشی، دوباره پرسش‌نامه عزت نفس Cooper-Smith را تکمیل نمودند. همچنین به منظور تحلیل داده‌ها از آزمون t همبسته استفاده شد. یافته‌ها: تحلیل داده‌های پژوهش نشان داد که آموزش مهارت‌های ارتباط مؤثر با افزایش عزت نفس در آزمودنی‌ها ارتباط معنی‌داری دارد. نتیجه گیری: نتایج حاصل از پژوهش نشان می‌دهد که آموزش مهارت‌های ارتباط مؤثر باعث افزایش عزت نفس دختران دچار ناتوانی جسمی- حرکتی می‌شود.

Title: مقایسه حداکثر گشتاور اكسنتريك عضلات ران و مچ در زنان ورزشكار با ناپایداری عملکردی مچ پا

http://jrrs.mui.ac.ir/index.php/jrrs/article/view/495

مقدمه: ناپایداری عملکردی مچ پا (Functional ankle instability یا FAI) به دنبال پیچ خوردگی خارجی مچ، شایع‌ترین آسیب ورزشی رشته‌های پرشی به حساب می‌آید. یکی از علل شیوع اسپرین مجدد مچ پا، ضعف عضلات مچ و مفاصل بالاتر گزارش شده است. هدف از تحقیق حاضر، بررسی حداکثر گشتاور اکسنتریک عضلات ران و مچ پا در ورزشکاران مبتلا به ناپایداری عملکردی مچ پا بوده است. مواد و روش‌ها: 30 نفر از زنان ورزشکار سالم و مبتلا به ناپایداری عملکردی مچ پا حاضر در لیگ برتر رشته‌های بسکتبال و والیبال تهران در این تحقیق شرکت نمودند. پس از انجام آزمون‌های بالینی و پر کردن پرسش‌نامه، آزمودنی‌ها به دو گروه شاهد و با ناپایداری عملکردی مچ پا تقسیم شدند. سپس نسبت حداکثر گشتاور اکسنتریک نرمال شده به وزن در حرکات اینورژن و اورژن مچ پا و ابداکشن و اداکشن ران به ترتیب با سرعت 60 و 90 درجه بر ثانیه تعیین شد. تجزیه و تحلیل داده‌ها با استفاده از نرم‌افزار13/5SPSS و آزمون آماری Independent t انجام شد (0/05 > P). یافته‌ها: تنها در نسبت حداکثر گشتاور اکسنتریک به وزن عضلات ابداکتور ران افراد مبتلا به ناپایداری عملکردی مچ پا و سالم تفاوت معنی‌دار وجود داشت. نتیجه گیری: مطالعه حاضر نشان داد یکی از عوارض ناپایداری عملکردی مچ پا ضعف عضلات ابداکتور ران است. بر این اساس، جامعه ورزشکاران مبتلا به ناپایداری عملکردی مچ پا باید به قدرت عضلات ابداکتور ران در برنامه توان‌بخشی توجه بیشتری نشان دهند.

Title: تأثير يك دوره تمرين در آب بر ویژگی‌های تعادلی و راه رفتن مردان سالمند سالم غیرفعال

http://jrrs.mui.ac.ir/index.php/jrrs/article/view/199

مقدمه: با بالا رفتن سن مشکلات نیز افزایش می‌یابد و سالمندان را در سطوح مختلف تحت تأثیر قرار می‌دهد. تحقیق حاضر با هدف مقایسه تأثیر تمرین در آب بر کنترل تعادل و راه‎ رفتن مردان سالمند انجام شد. مواد و روش‌ها: این تحقیق از نوع نیمه تجربی با طرح پیش آزمون- پس آزمون با یک گروه مداخله تمرینی و یک گروه شاهد بود. به همین منظور تعداد 30 نفر به صورت در دسترس از اماکن عمومی و پارک‎ها انتخاب و به روش تصادفی به دو گروه تجربی (با ميانگين و انحراف استاندارد سني 1/98 ± 68/53 سال، وزن 2/19 ± 69/45 کيلوگرم، قد 3/08 ± 168/46 سانتي‌متر) و شاهد (با ميانگين و انحراف استاندارد سني 2/87 ± 68/66 سال، وزن 3/13 ± 72/60 کيلوگرم، قد 3/41 ± 170/53 سانتي‌متر) تقسیم شدند. گروه تجربی به مدت 6 هفته، هر هفته سه جلسه و هر جلسه به مدت 60 دقیقه در تمرینات شرکت کردند. تغییرات در تعادل و راه رفتن آزمودنی‌ها، قبل و بعد از دوره تمرین به وسیله آزمون‌های ستاره، Sharpened-Romberg و Timed Up and Go اندازه‌گیری شد. نرمال بودن توزیع داده‌ها با استفاده از آزمون نرمالیته Kolmogorov–Smirnov و برابری واریانس‎ها با استفاده از آزمون Levine بررسی شد. سپس جهت تجزیه و تحلیل آماری، آزمون‎های One-way ANOVA و Mixed ANOVA استفاده شد. در ضمن تجزیه و تحلیل داده‌ها با استفاده از نرم‌افزار 16SPSS انجام گرفت و سطح معنی‌داري 0/05 در نظر گرفته شد. یافته‌ها: اعمال برنامه تمرین در محیط آب بر بهبود تعادل و توانایی و استقلال راه رفتن مردان سالمند تأثیر معنی‎داری داشت (0/05 < P). همچنین نتایج حاکی از تفاوت معنی‎داری بین داده‎های پس ‎آزمون آزمودنی‌های گروه تجربی و گروه‎ شاهد بود (0/05 < P). با وجود این تفاوت معنی‎داری بین نمرات پیش ‎آزمون و پس ‎آزمون گروه‎ شاهد مشاهده نشد (0/05 < P). نتیجه گیری: بر اساس یافته‎های این مطالعه، شاید به کارگیری تمرینات در آب می‎تواند عاملی تأثیرگذار در بهبود تعادل، سرعت و استقلال در راه رفتن سالمندان باشد.

Title: مقایسه عملکردهای شناختی بیماران مبتلا به پارکینسون با افراد سالم

http://jrrs.mui.ac.ir/index.php/jrrs/article/view/389

مقدمه: هر چند بیماری پارکینسون جزو اختلالات حرکتی طبقه‌بندی می‌شود، اما مشکلات شناختی نیز به کرات در این بیماری مشاهده می‌شود. هدف پژوهش حاضر مقایسه عملکردهای شناختی بیماران مبتلا به پارکینسون با افراد سالم بود. مواد و روش‌ها: مطالعه حاضر از نوع علی- مقایسه‌ای یا پس رویدادی بود. 35 بیمار پارکینسونی که ملاک‌های ورود را داشتند به پژوهشگران ارجاع داده شدند و به آزمون ارزیابی شناختی مونترآل و پرسش‌نامه جمعیت شناختی پاسخ دادند. 40 فرد سالم نیز که همه ملاک‌های ورود به جز ابتلا به بیماری پارکینسون را داشتند، انتخاب شدند و به همان مقیاس‌ها پاسخ دادند. داده‌ها با نرم‌افزار آماری 16SPSS و با استفاده از روش‌های آماری ضریب همبستگی و ANCOVA مورد تحلیل قرار گرفت. یافته‌ها: نمرات بیماران مبتلا به پارکینسون در زیر مقیاس‌های عملکرد دیداری- فضایی، عملکرد اجرایی، حافظه و توجه به طور معنی‌داری پایین‌تر از افراد سالم بود (0/05 > P). نتیجه‌گیری: نقایص شناختی یکی از مشکلات شایع در بیماری پارکینسون می‌باشد که با توجه به پاتوژنز بیماری قابل تبیین است. از آن جایی که این مشکلات می‌توانند در مراحل تشخیص، سیر و پیش آگهی بیماری و پاسخ به درمان تداخل کنند، بنابراین ضروری است که در ارزیابی‌های این بیماران مورد توجه قرار گیرند.

Title: مقایسه فعالیت عضلات اطرف زانو هنگام راه رفتن و دویدن در افراد با ناهنجاری واروس زانو و گروه شاهد

http://jrrs.mui.ac.ir/index.php/jrrs/article/view/359

مقدمه: تغییرات وضعیتی اندام تحتانی با اختلال عملکرد حرکتی و به ویژه راه رفتن همراه است. باوجود این که واروس زانو یکی از ناهنجاری‌های شایع اندام تحتانی است، اثر آن بر عملکرد حرکتی کمتر مورد توجه قرار گرفته است. هدف پژوهش حاضر، مقایسه فعالیت الکترومایوگرافی عضلات اطراف زانو (با تأکید بر پدیده هم انقباضی بین عضلات آگونیست و آنتاگونیست) هنگام راه رفتن و دویدن در افراد دارای واروس زانو در مقایسه با گروه همسالان نرمال بود. مواد و روش‌ها: این پژوهش یک مطالعه شبه تجربی با گروه‌های آزمون و شاهد بود. تعداد 16 دانش‌آموز پسر با میانگین سن 6/0 ± 5/15 سال، به طور در دسترس در دو گروه همسان شده واروس زانو و شاهد به طور مساوی تقسیم شدند. میزان هم انقباضی عمومی و هم انقباضی جهت‌دار عضلات مفصل زانو طی مراحل مختلف راه رفتن (مرحله انتقال وزن و مرحله مید استانس/پروپالشن) و مرحله استانس دویدن از طریق فعالیت الکترومایوگرافی عضلات ناحیه مفصل زانو بررسی شد. یافته‌ها: فعالیت الکترومایوگرافی عضلات ناحیه مفصل زانو در مرحله انتقال وزن هنگام راه رفتن حدود 18 درصد و طی مرحله استانس دویدن حدود 31 درصد در افراد دارای واروس زانو در مقایسه با گروه شاهد بیشتر بود، اما از لحاظ آماری اختلاف معنی‌دار نبود (به ترتیب: 52/0 = P و 55/0 = P). آزمودنی‌های گروه واروس زانو در مرحله انتقال وزن راه رفتن و مرحله استانس دویدن میزان هم انقباضی جهت‌دار بیش‌تری را در عضلات ناحیه خارجی مفصل زانو نسبت به عضلات ناحیه داخلی نشان دادند. این در حالی بود که بر عکس افراد گروه شاهد از هم انقباضی بیشتر عضلات ناحیه داخلی برخوردار بودند. نتیجه‌گیری: این نتایج تفاوت معنی‌داری از لحاظ آماری در میزان هم انقباضی عضلات بین افراد با واروس زانو و گروه شاهد در هنگام راه رفتن و دویدن نشان نداد. اما به نظر می‌رسد وجود تفاوت موجود در فعالیت الکترومایوگرافی برخی عضلات اندام تحتانی بین افراد مبتلا به دفورمیتی واروس زانو و گروه شاهد هنگام راه رفتن و دویدن کاربردهای کلینیکی داشته باشد.

Title: ويژگي‌هاي بيومکانيکي غضروف مفصلي تيبيا در مدل تجربي استئوآرتريت خرگوش

http://jrrs.mui.ac.ir/index.php/jrrs/article/view/238

مقدمه: ليگامنت متقاطع قدامي (ACL یا Anterior cruciate ligament) يکي از مهم‌ترين عوامل ثبات و کنترل حرکت در مفصل زانو مي‌باشد که آسيب آن عوارض جدي حرکتي براي فرد به دنبال دارد. مطالعه حاضر با هدف بررسی اثر قطع کامل اين ليگامنت در زانوي خرگوش بر خواص بافت شناسي و بيومکانيکي غضروف مفصل مبتلا انجام شد. مواد و روش‌ها: 10 سر خرگوش نر بالغ سفيد از نژاد Dutch در دو گروه تقسيم شدند. در گروه جراحي، ليگامنت متقاطع قدامي زانوي چپ حيوان با جراحي قطع شد. اطلاعات بافت شناسي، ويژگي‌هاي بيومکانيکي و ضخامت غضروف فمور و تيبياي زانوي چپ پس از 62 روز از انجام جراحي ثبت شد. در گروه سالم، حيوان در اين مدت تحت هيچ گونه مداخله‌اي قرار نگرفت. مشخصات بيومکانيکي پلاتوي داخلي تيبيا و کنديل داخلي فمور چپ در تست تنش- استراحت (Stress-relaxation) دو فازه (Biphasic) بررسي گرديد. با استفاده از داده‌هاي استخراج شده، حداکثر نيرو، ضريب الاستيک، نيروي تعادلي و ضريب تعادلي محاسبه گرديدند. مقايسه داده‌ها با آزمون Man-Withney U صورت گرفت. براي جلوگيري از خطاي ناشي از انجام تست بيومکانيکي، مطالعه بافت شناسي در پلاتوي خارجي تيبيا و کنديل خارجي فمور انجام شد. یافته‌ها: در گروه جراحي، غضروف مفصلي فمور و تيبياي چپ هر دو تخريب شدند؛ در حالي که تنها ضخامت غضروف فمور کاهش معني‌داري نسبت به گروه سالم نشان داد (0/009 = P). کليه پارامترهاي بيومکانيکي فمور در گروه جراحي نسبت به گروه سالم کاهش نشان دادند (0/05 > P). تنها استثنا در اين زمينه ضريب تعادلي (Aggregate modulus) بود که مقدار آن در دو گروه به طور تقريبی يکسان بود. مشخصات بيومکانيکي غضروف تيبيا تفاوت خاصي در دو گروه نشان نداد. نتیجه گیری: در 9 هفته اول پس از قطع کامل ليگامنت متقاطع قدامي، در حالي که شدت تغييرات ساختاري در غضروف مفصلي فمور تا حدودي کمتر از تيبيا است، تغييرات عملکردي اغلب در غضروف فمور مشاهده شد. اما غضروف تيبيا با وجود تخريب قابل ملاحظه، همچنان خواص بيومکانيکي خود را حفظ نمود. تغييرات گزارش شده در اين مقاله مي‌تواند مدرک مضاعفي باشد دال بر اينکه اين مدل حيواني، تغييرات خاص ناشي از استئوآرتريت را به خوبي نشان مي‌دهد.

Title: اثربخشی یک دوره آب درمانی و فیزیوتراپی بر عملکرد، راه رفتن و کنترل دینامیک افراد مبتلا به استئوآرتریت مفصل زانو

http://jrrs.mui.ac.ir/index.php/jrrs/article/view/158

مقدمه: یکی از روش‌های غیر دارویی که در درمان استئوآرتریت استفاده می‌شود، آب درمانی است. هدف از تحقیق حاضر بررسی و مقایسه اثربخشی یک دوره آب درمانی بر عملکرد، راه رفتن و کنترل دینامیک افراد مبتلا به استئوآرتریت مفصل زانو بود. مواد و روش‌ها: 36 بیمار مبتلا به استئوآرتریت زانو به طور تصادفی در سه گروه آب درمانی، فیزیوتراپی و شاهد طبقه‌بندی شدند. سپس آزمودنی‌های گروه آب درمانی و فیزیوتراپی به مدت 6 هفته برنامه آب درمانی و فیزیوتراپی مربوط به خود را دریافت نمودند. متغیرهای اندازه‌گیری شده شامل تست پله، تست Up & Go زمان‌بندی شده و تست شش دقیقه راه رفتن بودند که به ترتیب برای ارزیابی کنترل تعادل، عملکرد فیزیکی و راه رفتن در پیش آزمون و پس آزمون صورت گرفت. یافته‌ها: آب درمانی و فیزیوتراپی تعادل، راه رفتن و عملکرد فیزیکی را در بیماران مبتلا به استئوآرتریت زانو بهبود می‌بخشند (05/0 > P)، اما بین اثربخشی آب درمانی و فیزیوتراپی بر تعادل، راه رفتن و عملکرد فیزیکی آزمودنی‌های مبتلا به استئوآرتریت زانو تفاوت معنی‌داری وجود ندارد (05/0 < P). نتیجه گیری: با وجود اثربخشی هر دو روش آب درمانی و فیزیوتراپی بر عملکرد، راه رفتن و کنترل دینامیک افراد مبتلا به استئوآرتریت مفصل زانو، تفاوت معنی‌داری بین دو روش درمانی مشاهده نشد.

Title: تأثیر یک دوره تمرینات منتخب ورزش در آب بر درد، میزان علایم، عملکرد حرکتی و کیفیت زندگی زنان سالمند مبتلا به استئوآرتریت زانو

http://jrrs.mui.ac.ir/index.php/jrrs/article/view/444

مقدمه: امروزه شرکت در فعاليت بدني در پيش‌گيري از بیماری‌های مزمني همچون استئوآرتريت پيشنهاد می‌شود. هدف پژوهش حاضر، بررسی تأثیر یک دوره تمرینی ورزش در آب بر شدت درد زانو، ميزان علایم و عملكرد حركتي در فعالیت‌های روزانه، ورزشي- تفريحي و کيفيت زندگي زنان سالمند بود. مواد و روش‌ها: 12 زن سالمند مبتلا به استئوآرتریت زانو با ميانگين و انحراف استاندارد سن 4/70 ± 52 سال، قد 6/45 ± 163/17 سانتي‌متر، وزن 6/69 ± 67/83 کيلوگرم به صورت در دسترس هدفمند، به عنوان آزمودني انتخاب شدند. برای جمع‌آوری داده‌ها از پرسش‌نامه جهانی و بومی‌سازی شده پیامد صدمات زانو و استئوآرتریت استفاده شد. نمونه‌ها برنامه تمرینی ورزش در آب را به مدت 8 هفته زیر نظر مربی آب درمانی انجام دادند. براي تجزيه و تحليل داده‌ها از Dependent t در سطح معنی‌داري استفاده گردید (0/05 > P). یافته‌ها: شدت درد زانو (0/019 = P)، عملکرد حرکتی در فعالیت‌های روزانه (0/020 = P)، ورزشي- تفريحي (0/003 = P) و کیفیت زندگی (0/048 = P) پس از اجرای پروتکل به میزان معنی‌داری بهبود یافته بود، در حالی که بین پیش تا پس آزمون ميانگين میزان علایم (0/418 = P) تفاوت معني‌داري وجود نداشت. نتیجه گیری: نتايج نشان داد که برنامه تمرینی ورزش در آب می‌تواند به عنوان یک روش تمرینی ایمن و مؤثر در بهبود درد، عملکرد و کیفیت زندگی زنان سالمند داراي استئوآرتریت زانو مورد توجه قرار گیرد.

Title: آزمون سنجش اعتبار اندازه‌گیری نیروی واکنشی مفصل پتلوفمورال در وضعیت گوناگون چسب زنی مفصل پتلوفمورال حین انجام حرکت چمباتمه یک طرفه: آزمون مقدماتی

http://jrrs.mui.ac.ir/index.php/jrrs/article/view/449

مقدمه: ممکن است بتوان با اندازه‌گیری نیروهای واکنشی مفصل پتلوفمورال (Patellofemoral joint reaction force یا PFJRF)، شواهد معتبری را در خصوص نحوه اصلاح راستای بر هم خورده پتلا در مبتلایان به سندرم دردناک مفصل پتلوفمورال به دست آورد. هدف از مطالعه پیش رو سنجش میزان اعتبار اندازه‌گیری این نیروها در خلال شرایط مختلف مفصل پتلوفمورال حین حرکت چمباتمه بر روی افراد سالم بود. مواد و روش‌ها: با استفاده از سیستم تحلیل‌گر حرکتی و سکوی ثبت نیرو، نیروهای واکنشی وارده بر مفصل پتلوفمورال هشت داوطلب سالم در حین فعالیت چمباتمه یک طرفه مورد ارزیابی قرار گرفت. داده‌های پژوهش از نشان‌گرهای سطحی نصب شده بر مناطق انتخابی جمع‌آوری گردید. این فرایند بر روی پاهای راست کلیه داوطلبان قبل از انجام چسب زنی (Before taping یا BT)، در حین نصب چسب (During taping یا WT) و اندک زمان پس از اعمال چسب (Shortly after patellar taping یا SAT) بر روی پتلا به مورد اجرا در آمد. نیروهای مورد اشاره، با استفاده از مدل بیومکانیکی مفصل پتلوفمورال مورد محاسبه قرار گرفت. یافته‌ها: هیچ گونه اختلاف معنی‌داری بین میانگین (انحراف معیار) نیروهای واکنشی در سه وضعیت BT (25/455، 55/2100 نیوتن)، WT (45/516، 20/2026 نیوتن) و SAT (30/669، 35/2055 نیوتن) وجود نداشت (05/0 > P). مقادیر مربوط به ضریب تغییر (CV)، ضریب ارتباط درون گروهی (ICC)، حداقل اختلاف معنی‌دار (LSD) و خطای معیار اندازه‌گیری (SEM) همگی نشان دهنده اعتبار بالای اندازه‌گیری‌های میزان نیروهای وارده بر مفصل پتلوفمورال در حین فعالیت چمباتمه یک طرفه بود (05/0 > P). نتیجه‌گیری: اعتبار بالای اندازه‌گیری PFJRF نشان می‌دهد که در مطالعات آتی می‌توان بر این اندازه‌گیری‌ها در حین حرکت چمباتمه یک طرفه اتکا نمود.

Title: مقایسه اثر تقویت عضلات دور کننده و چرخاننده خارجی مفصل ران با عضلات چهارسر در بیماران مبتلا به سندرم درد کشککی- رانی

http://jrrs.mui.ac.ir/index.php/jrrs/article/view/319

مقدمه: سندرم درد کشککی– رانی یکی از اختلالات ارتوپدی زانو است که در سنین جوانی و بیشتر در زنان شایع می‌باشد. کاهش قدرت و کوتاه شدن زمان رفلکس عضله پهن مورب داخلی از یک طرف و کاهش قدرت عضلات دور کننده و چرخاننده خارجی استخوان ران که باعث افزایش زاویه Q می‌شود، از طرف دیگر به عنوان عوامل اصلی این سندرم شناخته شده‌اند. تحقیق حاضر مقایسه اثر تقویت عضلات دور کننده و چرخاننده خارجی ران را با عضلات چهارسر در بیماران مبتلا به سندرم درد کشککی- رانی بررسی نموده است. مواد و روش‌ها: 26 بیمار مبتلا به سندرم درد کشککی- رانی به صورت تصادفی در دو گروه یک و دو قرار گرفتند. گروه یک به مدت 8 هفته هر هفته سه روز، تمرینات قدرتی عضلات دور کننده و چرخاننده خارجی مفصل ران را انجام داد. گروه دوم در مدت مشابه تمرینات قدرتی عضلات چهارسر ران را با تأکید بر عضلات پهن مورب داخلی انجام داد. از دینامومتر دستی، پرسش‌نامه درد VAS (Visual analog scale) و عملکرد WOMAC (Western ontario and mcmaster university osteoarthritis)، قبل و بعد از دوره تمرین جهت اندازه‌گیری قدرت، درد و عملکرد استفاده شد. به منظور تجزیه و تحلیل داده‌ها از آزمون ANOVA برای اندازه‌های تکراری و از آمار توصیفی جهت بررسی اطلاعات توصیفی استفاده شد. یافته‌ها: هر دو گروه تفاوت معنی‌داری را قبل و بعد از مداخله در مقیاس درد VAS، مقیاس عملکرد WOMAC و قدرت عضلات دور کننده و چرخاننده خارجی در گروه یک و چهارسر رانی در گروه دو نشان دادند (01/0 ≤ P). مقایسه داده‌های بین دو گروه در مقیاس درد VAS و مقیاس عملکرد WOMAC تفاوت معنی‌داری را در قبل و پس از دوره تمرینی نشان نداد (01/0 ≤ P). نتیجه گیری: پس از 8 هفته تمرین درمانی، هر دو گروه کاهش درد و بهبود عملکرد و افزایش قدرت عضلات دور کننده و چرخاننده خارجی مفصل ران در گروه یک و قدرت عضلات چهارسر ران در گروه دو را نشان دادند. بر مبنای تحقیق حاضر تقویت عضلات مفصل ران و زانو می‌تواند در کاهش درد و افزایش عملکرد بیماران مبتلا به این سندرم مؤثر باشد.

Title: مطالعه تأثیر ماساژ سوئدی و حرکت درمانی بر سندرم درد کشککی- رانی

http://jrrs.mui.ac.ir/index.php/jrrs/article/view/293

مقدمه: سندرم درد کشککی- رانی یکی از شایع‌ترین اختلالات مفصلی است که با درد جلوی زانو و ضعف عضله پهن داخلی مایل شناخته می‌شود. هدف از این مطالعه، بررسی و مقایسه تأثیر ماساژ سوئدی و تمرینات کششی- تقویتی بر علایم این بیماران بود. مواد و روش‌ها: تعداد 36 بیمار مرد مبتلا به سندرم درد کشککی- رانی به صورت تصادفی در سه گروه ماساژ، حرکت درمانی و شاهد قرار گرفتند. دو گروه تجربی اول به مدت 8 هفته پروتکل درمانی مربوط به خود را دریافت نمودند، در حالی که گروه شاهد هیچ گونه برنامه درمانی را دنبال نکرد. از مقیاس بصری سنجش درد در حالت استراحت و آزمون 6 دقیقه راه رفتن به ترتیب جهت اندازه‌گیری میزان درد و ارزیابی عملکرد آزمودنی‌ها پیش و پس از اعمال مداخله درمانی استفاده شد. یافته‌ها: میزان درد در دو گروه تحت درمان با ماساژ سوئدی و تمرینات تقویتی– کششی نسبت به گروه شاهد به طور معنی‌داری کاهش یافت (0/001 > P)، در حالی که این میزان کاهش درد در آزمودنی‌های گروه ماساژ درمانی به طور معنی‌داری بیشتر از گروه حرکت درمانی بود (0/001 > P). هم چنین مسافت طی شده در مدت زمان 6 دقیقه، در بیماران هر دو گروه ماساژ سوئدی و تمرین درمانی به طور معنی‌داری نسبت به گروه شاهد افزایش پیدا کرد (0/001 > P)، ولی تفاوت معنی‌داری بین گروه ماساژ درمانی و گروه حرکت درمانی وجود نداشت (0/05 < P).  نتیجه گیری: بر اساس یافته‌های پژوهش حاضر می‌توان نتیجه گرفت که ماساژ سوئدی و تمرینات تقویتی– کششی اثرات مشابهی بر علایم سندرم درد کشککی- رانی دارند، اگر چه ماساژ نسبت به حرکت درمانی بیشتر باعث کاهش درد این بیماران می‌شود.

Title: اثر دو شدت حسی و حرکتی تحریک الکتریکی کاتدی بر پتانسیل آسیب و ویژگی‌های بیومکانیکی زخم حاد تمام ضخامت پوستی در موش صحرایی

http://jrrs.mui.ac.ir/index.php/jrrs/article/view/258

مقدمه: هدف این مطالعه بررسی اثر شدت جریان حسی و حرکتی تحریک الکتریکی کاتدی بر پتانسیل آسیب، بسته شدن سطح زخم و ویژگی‌های بیومکانیکی بافت ترمیم شده بود. مواد و روش‌ها: 30 سر موش صحرایی نر بالغ نژاد اسپراگ دالی با وزن 400-300 گرم به صورت تصادفی در سه گروه شاهد، تحریک شدت حسی و شدت حرکتی قرار گرفتند. برش تمام ضخامت پوستی به طول 5/2 سانتی‌متر در ناحیه پشتی حیوان ایجاد شد. در گروه تحریک حسی، جریان مستقیم با شدت 600 میکروآمپر و در گروه تحریک حرکتی، جریان تک فاز با پالس‌های مربعی یک سویه، عرض پالس 300 میکروثانیه و شدت 3/2-2/5 میلی‌آمپر به مدت یک ساعت و یک روز در میان تا 21 روز اعمال گردید. سطح زخم و پتانسیل تفاضلی پوست از روز اول و سپس یک روز در میان اندازه‌گیری شد. در روز 21 تست کششی یک محوری انجام شد و تنش، کرنش، مدول الاستیک، و سطح زیر منحنی نیرو- تغییر شکل محاسبه شد. یافته‌ها: پتانسیل پوست در گروه تحریک حسی و شاهد در روز 15 و در گروه تحریک حرکتی در روز 17 به سطح طبیعی رسید. سطح زخم در گروه تحریک حسی در روز 15 و در گروه تحریک حرکتی و شاهد در روز 17 ترمیم به طور کامل بسته شد. تفاوت معنی‌داری در پارامترهای بیومکانیکی بافت ترمیم شده بین سه گروه مشاهده نشد (0/05 > P). نتیجه گیری: شدت جریان حرکتی بر خلاف تصور، تأثیر بیشتری بر پارامترهای بیومکانیکی بافت ترمیم شده نداشت. به نظر می‌رسد تغییر محیط مکانیکی ایجاد شده با اعمال تحریک الکتریکی، نتوانسته است نقش تنش‌ها و کرنش‌های طبیعی روزانه را که به بافت وارد می‌شود، بازسازی نماید و موجب بلوغ زودهنگام فیبرهای کلاژن و پیوندهای درون و بین مولکولی شود.

Title: معرفي كاربرد فشار هوای مثبت مداوم (CPAP) در درمان پرخيشومي: ارایه گزارش مورد

http://jrrs.mui.ac.ir/index.php/jrrs/article/view/395

مقدمه: دکتر David Kuehn در سال 1991 از فشار هوای مثبت مداوم (Continuous positive air pressure یا CPAP) برای درمان بیماران با پرخیشومی خفیف و گپ کمتر از mm 2 استفاده کرد. هدف از این مطالعه موردی ارایه نتایج درمان CPAP بر روی یک بیمار فارسی زبان با پرخیشومی خفیف بود. مواد و روش‌ها: بیمار یک نوجوان 12 ساله بود. شکایت عمده خانواده از گفتار نامفهوم و پرخیشومی او بود که بعد از جراحی لوزه سوم بدتر شده بود. بیمار 2 ماه گفتاردرمانی مستقیم را بدون هیچ گونه نتیجه‌ای دریافت کرده بود. بعد از معاینه توسط آسیب‌شناس گفتار و زبان، انجام آندوسکوپی ضروری تشخیص داده شد. مشاهده آندوسکوپی بیمار نشان داد که گپ موجود در دریچه کامی- حلقی (Velopharyngeal یا VP) کمتر از mm 2 می‌باشد و حرکت نرمکام و دیواره‌های طرفی حلق به نسبت خوب و بر اساس ارزیابی ادراکی گفتار پرخیشومی خفیف بود. با توجه به مشخصه‌های گفتاری و آناتومیکی، درمان با استفاده از CPAP بهترین روش درمانی تشخیص داده شد. بیمار 8 هفته درمان CPAP را مطابق با برنامه تعیین شده دریافت کرد. یافته‌ها: در آندوسکوپی بعد از درمان هیچ گونه گپی مشاهده نشد و گفتار پرخیشومی و نامفهوم بیمار به طور کامل درمان شده بود. نتیجه گیری: اگر یادگیری نامناسب یک علت اصلی در گفتار پرخیشومی تصور شود، این فرضیه کاملاً معقول است که بازخورد اطلاعات به فرد بیمار تأثیر به سزایی در درمان مشکل وی خواهد داشت. CPAP نیروی محرک قوی را برای تنظیم فشار هوا در مسیر صوتی ایجاد می‌کند. این نیرو یک تعامل مستقیم با فشار مثبت تولید شده به وسیله مکانسیم تنفسی در طی گفتار دارد. یافته‌های پژوهشی نشان داده است که درمان CPAP به بهتر بسته شدن دریچه VP در گفتار پیوسته کمک می‌کند که این عمل به دلیل تقویت عضلات دریچه VP اتفاق می‌افتد.
